# Supplementary material for: Left-handed DNA for efficient highly multiplexed imaging at single-protein resolution
Source: Nat Commun. 2025 Oct 2;16:8773. doi: 10.1038/s41467-025-64228-x (PMC12491495; doi:10.1038/s41467-025-64228-x)
Supplement: Supplementary file 1 — Supplementary Information [file 41467_2025_64228_MOESM1_ESM.pdf]

Supplementary Information

Left-handed DNA for efficient highly multiplexed imaging at single-protein resolution

Eduard M. Unterauer[1,2,*], Eva-Maria Schentarra[1,2,*], Isabelle Pachmayr[1,3,*], Taisha Tashrin[2,*], Jisoo Kwon[2], Sebastian Strauss[2], Kristina Jevdokimenko[4], Rafal Kowalewski[1,2], Felipe Opazo[4,5,6], Eugenio F. Fornasiero[4,7], Luciano A. Masullo[1,**], Ralf Jungmann[1,2,**]

[1]Faculty of Physics and Center for Nanoscience, Ludwig Maximilian University, Munich, Germany. [2]Max Planck Institute of Biochemistry, Martinsried, Germany. [3]Department of Chemistry and Biochemistry, Ludwig Maximilian University, Munich, Germany. [4]Institute of Neuro- and Sensory Physiology, University Medical Center Göttingen, Göttingen, Germany. [5]Center for Biostructural Imaging of Neurodegeneration (BIN), University Medical Center Göttingen, Göttingen, Germany. [6]NanoTag Biotechnologies GmbH, Göttingen, Germany. [7]Department of Life Sciences, University of Trieste, Trieste, Italy.
[*]These authors contributed equally.
[**]Correspondence should be addressed to masullo@biochem.mpg.de or jungmann@biochem.mpg.de.

**Supplementary Figure 1**	**5-nm DNA Origami demonstration and comparison**
**Supplementary Figure 2**	**Kinetics comparison of L and R sequences on DNA origami**
**Supplementary Figure 3**	**Kinetics comparison of L and R sequences on the nuclear pore complex**
**Supplementary Figure 4**	**Gallery of a 13-plex neuron atlas for the bottom-left field of view within the 200 x 200 µm² 4x4 grid**
**Supplementary Figure 5**	**Gallery of a 13-plex neuron atlas for the top-left field of view within the 200 x 200 µm² 4x4 grid**
**Supplementary Figure 6**	**Gallery of a 13-plex neuron atlas for the top-right field of view within the 200 x 200 µm² 4x4 grid**
**Supplementary Figure 7**	**Gallery of a 13-plex neuron atlas for the bottom-right field of view within the 200 x 200 µm² 4x4 grid**
**Supplementary Figure 8**	**Overlay of three separate Anion exchange chromatograms of DNA, unconjugated and DNA-conjugated GFP-Nb**
**Supplementary Table 1**	**Imager sequences**
**Supplementary Table 2**	**Handle sequences**
**Supplementary Table 3**	**Secondary barcode sequences**
**Supplementary Table 4**	**Sequence kinetics DNA origami (median values)**
**Supplementary Table 5**	**Sequence kinetics nuclear pore (median values)**
**Supplementary Table 6**	**List of affinity reagents with respective concentrations and dilutions**
**Supplementary Table 7**	**Experimental parameters for 13-plex neuronal atlas**
**Supplementary Table 8**	**Localization precision for 13-plex neuronal atlas**
**Supplementary Table 9**	**Comparison of current multiplexing approaches**
**Supplementary Table 10**	**Imaging parameters**

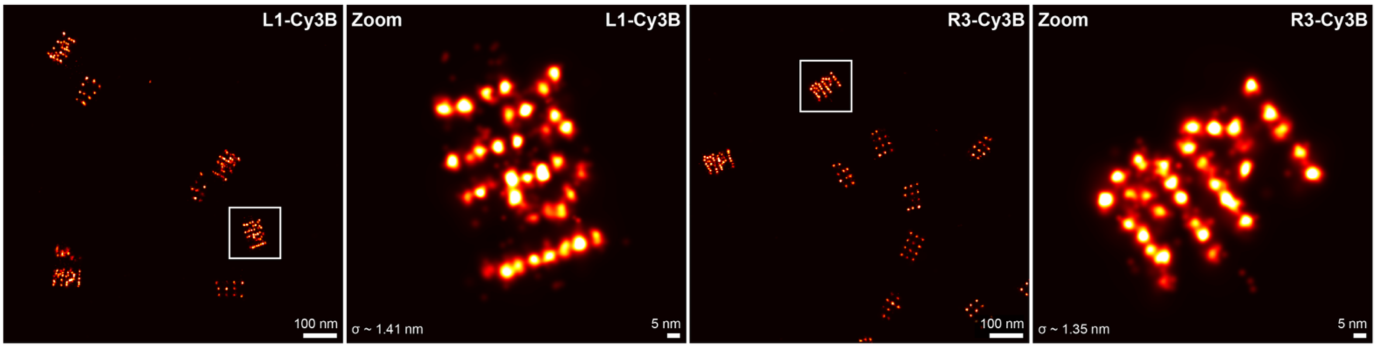

Supplementary Figure 1 | 5-nm DNA Origami demonstration and comparison. State of the art DNA-PAINT imaging with sub-5-nm localization precision measurements of L1 and R3.

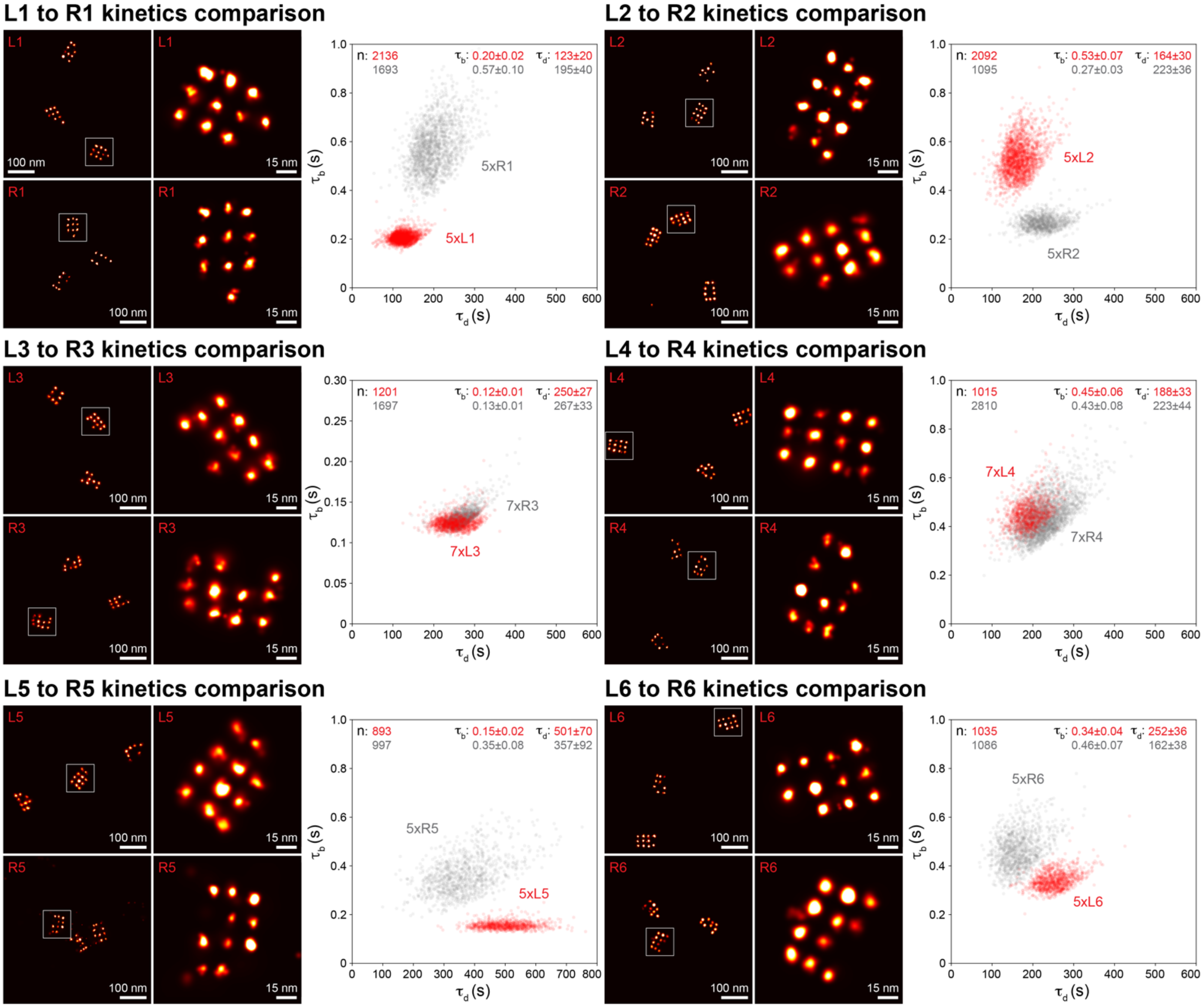

Supplementary Figure 2 | Kinetics comparison of L and R sequences on DNA origami. Pairwise comparisons of left-handed and right-handed sequences with an exemplary imaging result and the respective bright and dark times and DNA origami. Left shows an intermediate zoom-in with several 15-nm spaced DNA origami, while the middle shows a zoom-in into an individual DNA origami. Right shows a scatterplot of bright time vs dark time, with the number of binding sites included in the analysis as well as the fitted mean and standard deviation.

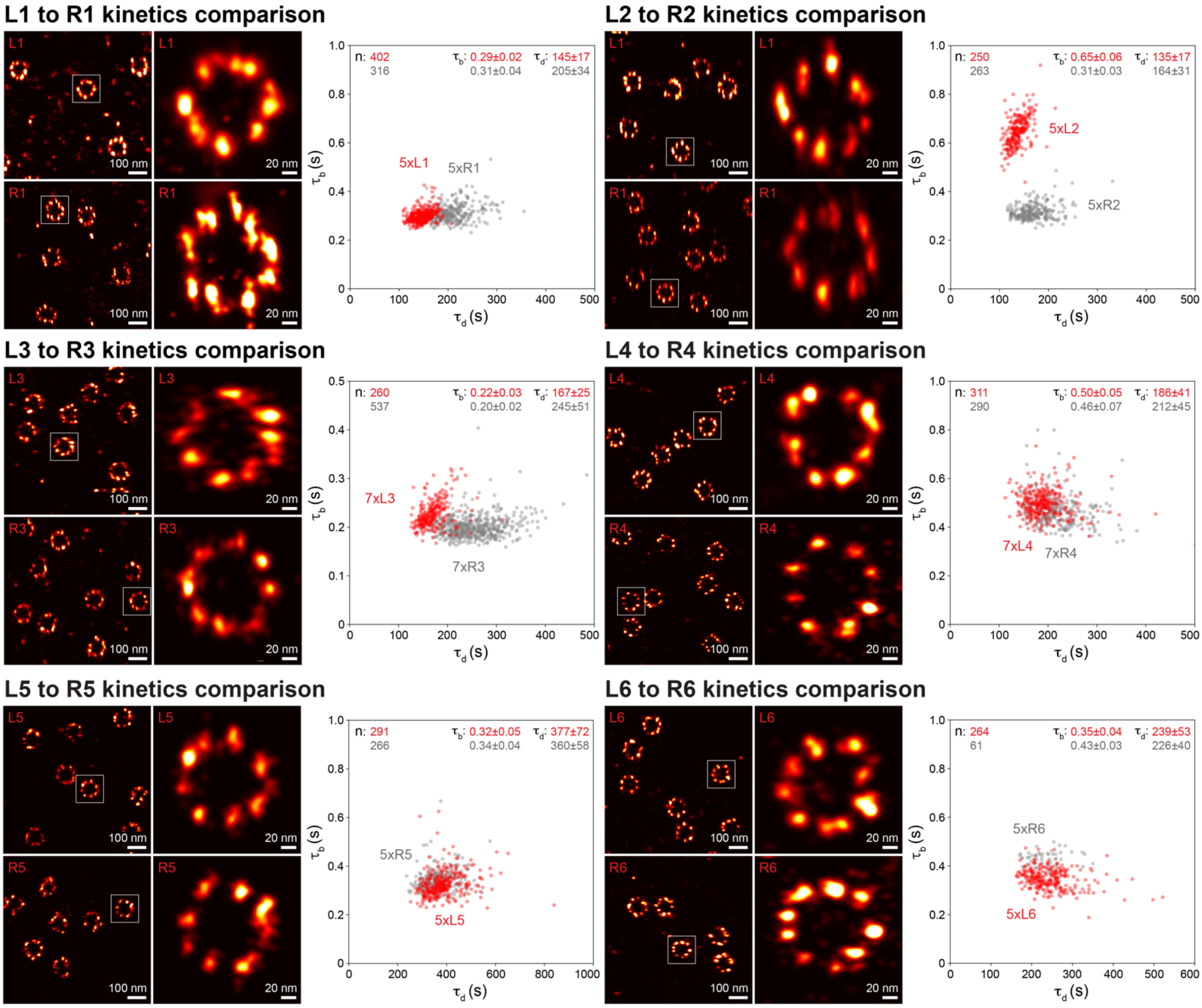

Supplementary Figure 3 | Kinetics comparison of L and R sequences on the nuclear pore complex. Pairwise comparisons of left-handed and right-handed sequences with an exemplary imaging result and the respective bright and dark times on the nuclear pore protein NUP96. Left shows an intermediate zoom-in with several nuclear pores, while the middle shows a zoom-in into an individual nuclear pore. Right shows a scatterplot of bright time vs dark time, with the number of binding sites included in the analysis as well as the fitted mean and standard deviation.

13-plex neuron atlas of a single field of view (bottom-left)

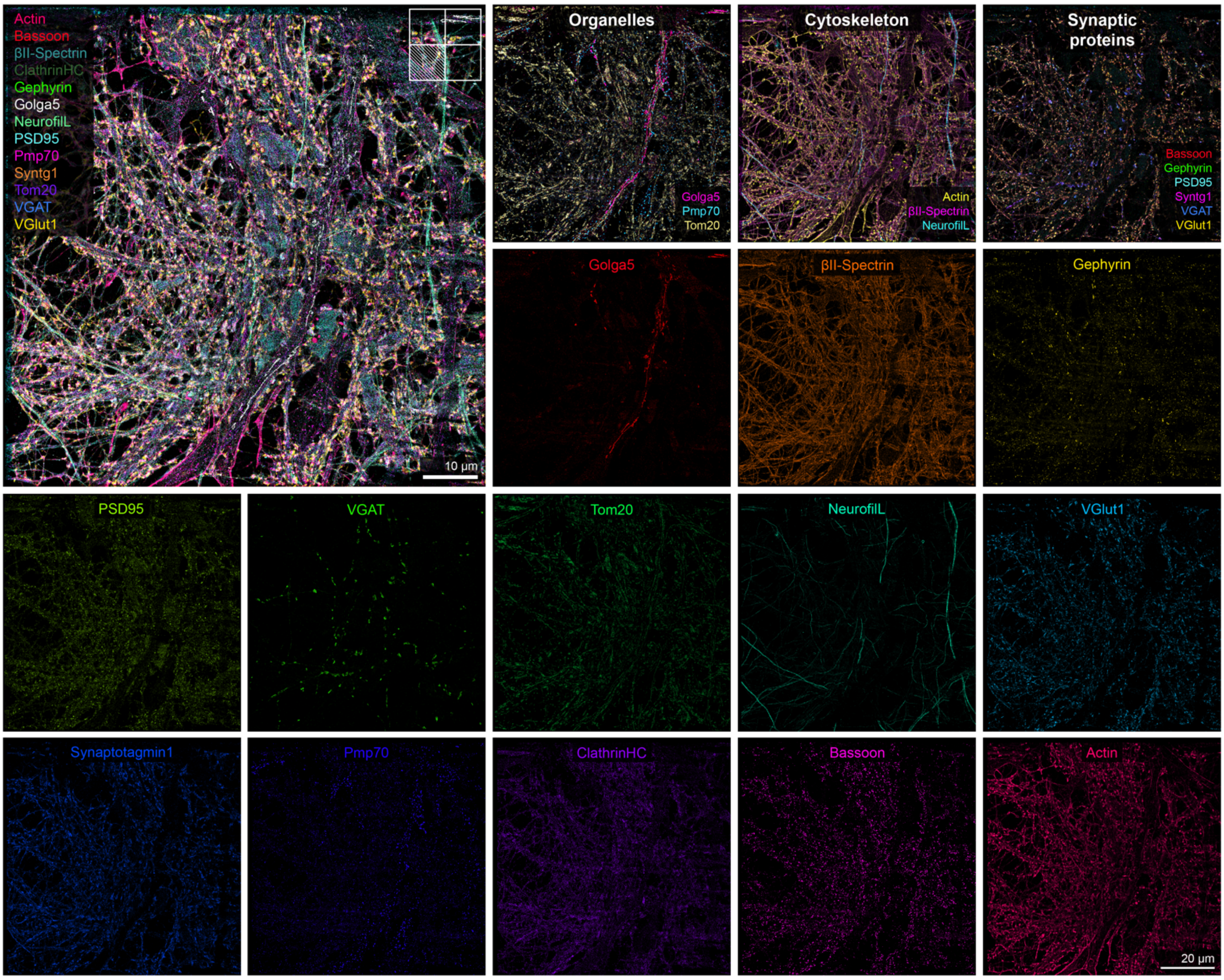

Supplementary Figure 4 | Supplementary Figure 4. Gallery of a 13-plex neuron atlas for the bottom-left field of view within the 200 x 200 µm² 4x4 grid. Multiplexed overlay image of 13 protein targets visualized simultaneously at single-protein resolution. The top row presents separate overlays for subsets of three organelle proteins (left), three cytoskeletal proteins (middle), and six synaptic proteins (right) from the same acquisition. Individual visualization of 13 protein targets includes synaptic scaffolding proteins (Bassoon, Gephyrin, PSD95), synaptic vesicle pool markers (VGlut1, VGAT, Synaptotagmin1), organelle markers (Tom20, Pmp70, Golga5), cytoskeletal proteins (βII-Spectrin, Neurofilament L, Actin), and an endocytic vesicle marker (Clathrin Heavy Chain). DNA-PAINT imaging rounds were completed in 10 minutes per target, achieving an average localization precision of 11.75 nm.

13-plex neuron atlas of a single field of view (top-left)

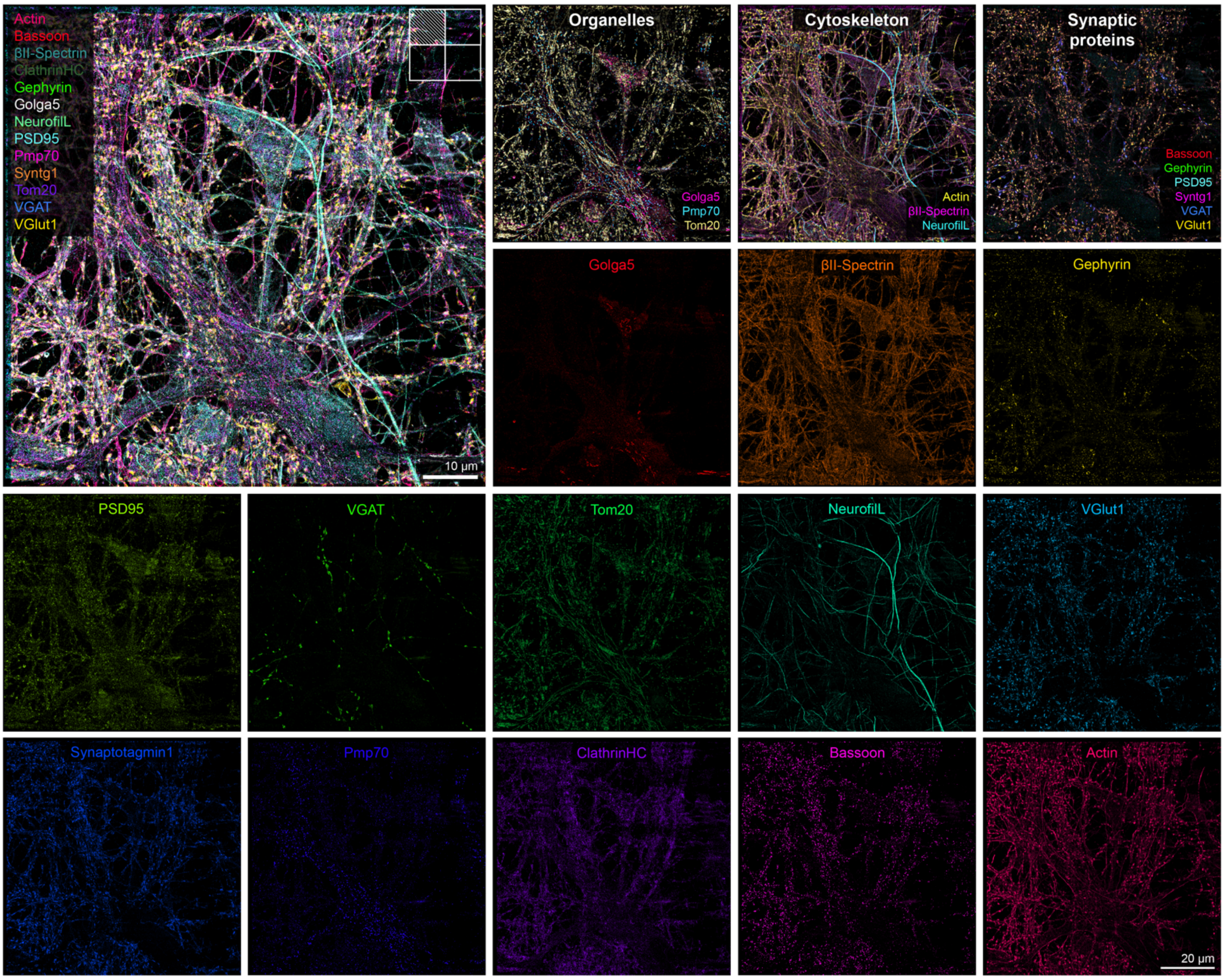

Supplementary Figure 5 | Gallery of a 13-plex neuron atlas for the top-left field of view within the 200 x 200 µm² 4x4 grid. Multiplexed overlay image of 13 protein targets visualized simultaneously at single-protein resolution. The top row presents separate overlays for subsets of three organelle proteins (left), three cytoskeletal proteins (middle), and six synaptic proteins (right) from the same acquisition. Individual visualization of 13 protein targets includes synaptic scaffolding proteins (Bassoon, Gephyrin, PSD95), synaptic vesicle pool markers (VGlut1, VGAT, Synaptotagmin1), organelle markers (Tom20, Pmp70, Golga5), cytoskeletal proteins (βII-Spectrin, Neurofilament L, Actin), and an endocytic vesicle marker (Clathrin Heavy Chain). DNA-PAINT imaging rounds were completed in 10 minutes per target, achieving an average localization precision of 11.75 nm.

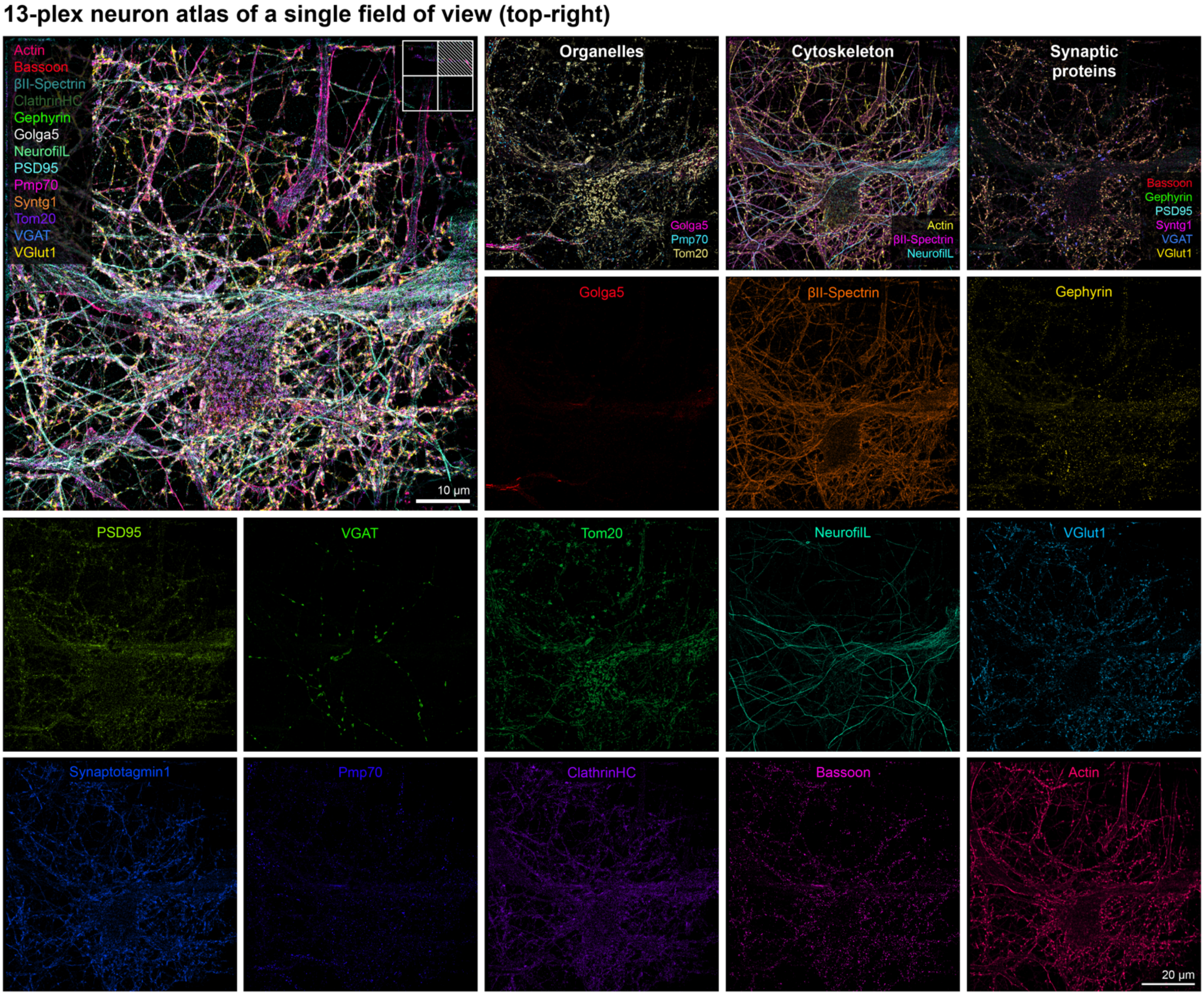

Supplementary Figure 6 | Gallery of a 13-plex neuron atlas for the top-right field of view within the 200 x 200 µm² 4x4 grid. Multiplexed overlay image of 13 protein targets visualized simultaneously at single-protein resolution. The top row presents separate overlays for subsets of three organelle proteins (left), three cytoskeletal proteins (middle), and six synaptic proteins (right) from the same acquisition. Individual visualization of 13 protein targets includes synaptic scaffolding proteins (Bassoon, Gephyrin, PSD95), synaptic vesicle pool markers (VGlut1, VGAT, Synaptotagmin1), organelle markers (Tom20, Pmp70, Golga5), cytoskeletal proteins (βII-Spectrin, Neurofilament L, Actin), and an endocytic vesicle marker (Clathrin Heavy Chain). DNA-PAINT imaging rounds were completed in 10 minutes per target, achieving an average localization precision of 11.75 nm.

13-plex neuron atlas of a single field of view (bottom-right)

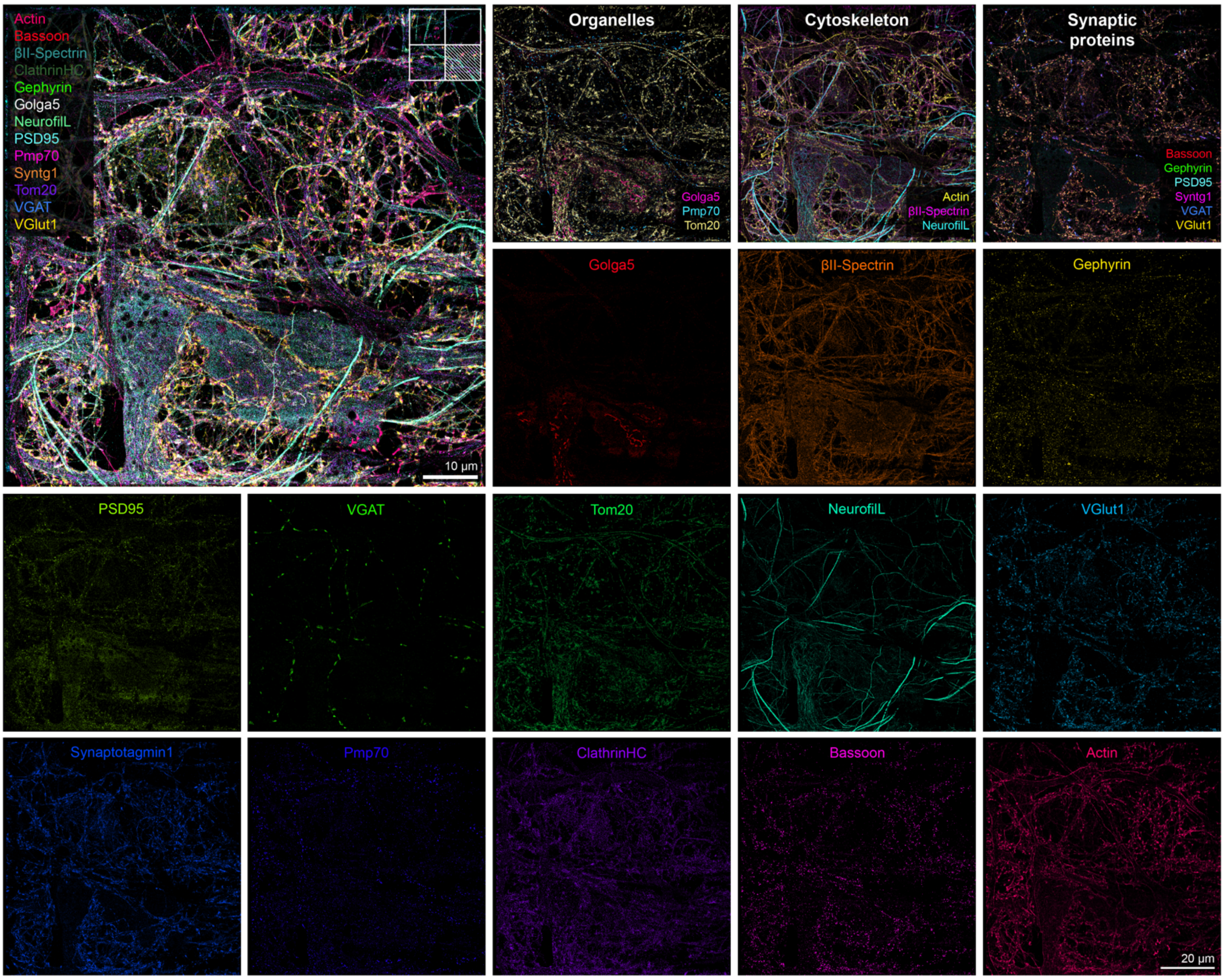

Supplementary Figure 7 | Gallery of a 13-plex neuron atlas for the bottom-right field of view within the 200 x 200 µm² 4x4 grid. Multiplexed overlay image of 13 protein targets visualized simultaneously at single-protein resolution. The top row presents separate overlays for subsets of three organelle proteins (left), three cytoskeletal proteins (middle), and six synaptic proteins (right) from the same acquisition. Individual visualization of 13 protein targets includes synaptic scaffolding proteins (Bassoon, Gephyrin, PSD95), synaptic vesicle pool markers (VGlut1, VGAT, Synaptotagmin1), organelle markers (Tom20, Pmp70, Golga5), cytoskeletal proteins (βII-Spectrin, NeurofilamentL, Actin), and an endocytic vesicle marker (Clathrin Heavy Chain). DNA-PAINT imaging rounds were completed in 10 minutes per target, achieving an average localization precision of 11.75 nm.

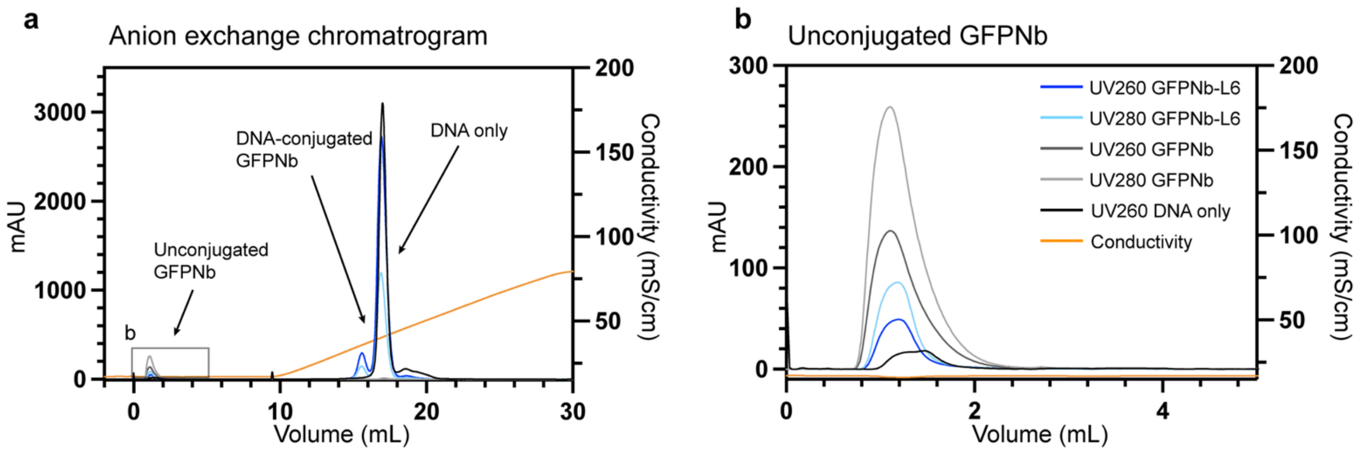

Supplementary Figure 8 | Overlay of three separate Anion exchange chromatograms of DNA, unconjugated and DNA-conjugated GFP-Nb. a, Flow through and wash steps for each run are characterized by low salt buffers, i.e. low conductivity (orange, 0-10 mL). Only the negatively charged DNA and DNA-conjugated proteins bind to the Anion exchange column. Thus, the unconjugated GFP-Nb is detected in the flow through (FT) (0-2 mL) of both the GFP-Nb only (UV260: dark gray, UV280: light gray) and the GFPNb-L6 (UV260: dark blue, UV280: light blue) sample. Bound DNA-conjugated GFP-Nb and DNA are eluted from the Anion exchange column by increasing conductivity, starting from 10 mL. The first elution peak represents the DNA-conjugated GFP-Nb, since this peak is not detected in the DNA only and the GFP-Nb only chromatogram. This peak is pooled for further use in DNA-PAINT microscopy. The free DNA elutes at higher conductivity than the DNA-conjugated GFP-Nb, as this is the only peak detected in the DNA only chromatogram (UV260: black). **b**, Zoom in of the flow-through of all three chromatograms. Unconjugated GFP-Nb does not bind to the column and is detected in the flow through. In a and b, mAU corresponds to milli-Absorbance Units, 1.0 AU equals a tenfold decrease in transmitted light intensity.

Supplementary Table 1 | Imager sequences

Imager name	Sequence	5'-mod	3'-mod	Vendor
R1	AGGAGGA	None	Cy3B	Metabion
L1	AGGAGGA*	None	Cy3B	Biomers
R2	GGTGGT	None	Cy3B	Metabion
L2	GGTGGT*	None	Cy3B	Biomers
R3 short	AGAGAGA	None	Cy3B	Metabion
R3 long	GAGAGAG	None	Cy3B	Metabion
L3	AGAGAGA*	None	Cy3B	Biomers
R4	TGTGTGT	None	Cy3B	Metabion
L4	TGTGTGT*	None	Cy3B	Biomers
R5	GAAGAAG	None	Cy3B	Metabion
L5	GAAGAAG*	None	Cy3B	Biomers
R6	TTGTTGTT	None	Cy3B	Metabion
L6	TTGTTGTT*	None	Cy3B	Biomers

*left-handed

Supplementary Table 2 | Handle sequences

Handle Name	Sequence	5'-mod	3'-mod	Vendor
R1	TCCTCCTCCTCCTCCTCCT	C-3 azide or Barcode sequence	None	Metabion
L1	TCCTCCTCCTCCTCCTCCT*	C-3 azide or Barcode sequence	None	Metabion
R2	ACCACCACCACCACCACCA	C-3 azide or Barcode sequence	None	Metabion
L2	ACCACCACCACCACCACCA*	C-3 azide or Barcode sequence	None	Metabion
R3	CTCTCTCTCTCTCTCTCTC	C-3 azide or Barcode sequence	None	Metabion
L3	CTCTCTCTCTCTCTCTCTC*	C-3 azide or Barcode sequence	None	Metabion
R4	ACACACACACACACACACA	C-3 azide or Barcode sequence	None	Metabion
L4	ACACACACACACACACACA*	C-3 azide or Barcode sequence	None	Metabion
R5	CTTCTTCTTCTTCTTCTTC	C-3 azide or Barcode sequence	None	Metabion
L5	CTTCTTCTTCTTCTTCTTC*	C-3 azide or Barcode sequence	None	Metabion
R6	AACAACAACAACAACAACAA	C-3 azide or Barcode sequence	None	Metabion
L6	AACAACAACAACAACAACAA*	C-3 azide or Barcode sequence	None	Metabion
1ry BC 13	TAAGATACGTCGACCCGATT	C-3 azide	None	Metabion
1ry BC 14	TAAACCCGCGTACCTCGATT	C-3 azide	None	Metabion

*left-handed

Supplementary Table 3 | Secondary barcode sequences

Barcode name	Handle name	Barcode Sequence	Readout Sequence	Vendor
BC1	R1	TGGTACTACGGGCGTATTGT	TCCTCCTCCTCCTCCTCCT	Metabion
BC2	L1	GGAAGATCGGACGAACCTAA	TCCTCCTCCTCCTCCTCCT*	Biomers
BC3	R2	TCACACGTCCGAGCACCAAT	ACCACCACCACCACCACCA	Metabion
BC4	L2	CAGCAGGCTCACCCGTTATA	ACCACCACCACCACCACCA*	Biomers
BC5	R3	ATGCTAAGCTGAGTTATAGT	CTCTCTCTCTCTCTCTCTC	Metabion
BC6	L3	GGACGTAATTCGCCGGTTTA	CTCTCTCTCTCTCTCTCTC*	Biomers
BC7	R4	ATAAACGGTCCGTTCGACTT	ACACACACACACACACACA	Metabion
BC8	L4	TTGAGAATAAGTGACCTCAT	ACACACACACACACACACA*	Biomers
BC9	R5	CTGAGAGGAATGACTCACTC	CTTCTTCTTCTTCTTCTTC	Metabion
BC10	L5	GTATACGTCGCGATATGAAT	CTTCTTCTTCTTCTTCTTC*	Biomers
BC11	R6	GCTCGTTTTACTAGTGAGGT	AACAACAACAACAACAACAA	Metabion
BC12	L6	TTTGCGGTACACAGCTACCA	AACAACAACAACAACAACAA*	Biomers
BC13	R3	AATCGGGTCGACGTATCTTA	CTCTCTCTCTCTCTCTCTC	Metabion
BC14	R2	AATCGAGGTACGCGGGTTTA	ACCACCACCACCACCACCA	Metabion

*left-handed

Supplementary Table 4 | Sequence kinetics DNA origami (median values)

Imager Name	n (DNA Origami)	τ_B±std (DNA Origami)	τ_D±std (DNA Origami)	k_{on} (DNA Origami)
R1	1693	0.57±0.10 s	194.8±40.0 s	51.3 * 10^6 $(Ms)^{-1}$
L1	2136	0.20±0.02 s	123.1±20.4 s	81.2 * 10^6 $(Ms)^{-1}$
R2	1095	0.27±0.03 s	222.8±35.6 s	44.9 * 10^6 $(Ms)^{-1}$
L2	2092	0.53±0.07 s	164.3±30.0 s	60.9 * 10^6 $(Ms)^{-1}$
R3	1697	0.13±0.01 s	266.8±27.2 s	37.5 * 10^6 $(Ms)^{-1}$
L3	1201	0.12±0.01 s	249.6±33.5 s	40.1 * 10^6 $(Ms)^{-1}$
R4	2810	0.43±0.07 s	223.4±43.8 s	44.8 * 10^6 $(Ms)^{-1}$
L4	1015	0.45±0.06 s	188.5±32.5 s	53.1 * 10^6 $(Ms)^{-1}$
R5	997	0.35±0.08 s	357.5±92.4 s	28.0 * 10^6 $(Ms)^{-1}$
L5	893	0.15±0.02 s	500.8±69.6 s	20.0 * 10^6 $(Ms)^{-1}$
R6	1086	0.46±0.07 s	162.1±38.3 s	61.7 * 10^6 $(Ms)^{-1}$
L6	1035	0.34±0.04 s	251.8±35.6 s	39.7 * 10^6 $(Ms)^{-1}$

Supplementary Table 5 | Sequence kinetics nuclear pore (median values)

Imager Name	n (Nuclear Pore)	τ_B±std (Nuclear Pore)	τ_D±std (Nuclear Pore)	k_{on} (Nuclear Pore)
R1	316	0.31±0.04 s	204.9±34.4 s	48.8 * 10^6 $(Ms)^{-1}$
L1	402	0.29±0.02 s	145.2±16.6 s	68.9 * 10^6 $(Ms)^{-1}$
R2	263	0.31±0.03 s	164.2±30.8 s	60.9 * 10^6 $(Ms)^{-1}$
L2	250	0.65±0.06 s	135.0±17.0 s	74.1 * 10^6 $(Ms)^{-1}$
R3	537	0.20±0.02 s	245.1±51.0 s	40.8 * 10^6 $(Ms)^{-1}$
L3	260	0.22±0.03 s	167.2±25.1 s	59.8 * 10^6 $(Ms)^{-1}$
R4	290	0.46±0.07 s	212.1±44.9 s	47.2 * 10^6 $(Ms)^{-1}$
L4	311	0.50±0.05 s	186.4±40.7 s	53.6 * 10^6 $(Ms)^{-1}$
R5	266	0.33±0.04 s	360.0±57.7 s	27.8 * 10^6 $(Ms)^{-1}$
L5	291	0.32±0.05 s	376.9±71.6 s	26.5 * 10^6 $(Ms)^{-1}$
R6	61	0.43±0.03 s	226.1±39.7 s	44.2 * 10^6 $(Ms)^{-1}$
L6	264	0.35±0.04 s	238.9±53.3 s	41.9 * 10^6 $(Ms)^{-1}$

Supplementary Table 6 | List of affinity reagents with respective concentrations and dilutions

Antibody/Nanobody	Vendor	Catalog Number	Stock-Concentration	Dilution
GFP	Nanotag	Cat# N0301	5 µM	1:200
Rabbit IgG	Nanotag	Cat# N2405	5 µM	-
Mouse kappa light chain	Nanotag	Cat# N1205	5 µM	-
Mouse monoclonal anti- α-Tubulin	Sigma-Aldrich	Cat# T6199; RRID:AB_477583	1.0 -1.2 mg/mL	1:200
Mouse monoclonal anti-Bassoon	Enzo Life Sciences	Cat# ADI-VAM-PS003-F; RRID:AB_1118105	1 mg/mL	1:200
Mouse monoclonal anti-Gephyrin	Synaptic Systems	Cat#147 011; RRID:AB_887717	1 mg/mL	1:200
Rabbit polyclonal anti-VGAT	invitrogen	Cat#PA5-27569; RRID:AB_2545045	1.46 mg/mL	1:250
Rabbit monoclonal anti-Tom20	Abcam	Cat#ab186735; RRID:AB_2889972	0.94 mg/mL	1:200
Rabbit polyclonal anti-VGlut1	Synaptic Systems	Cat#135303; RRID:AB_887875	1 mg/mL	1:200
Rabbit polyclonal anti-PMP70	Abcam	Cat#ab85550; RRID:AB_10672335	0.8 - 1 mg/mL	1:200
Mouse monoclonal anti-Neurofilament L	Synaptic Systems	Cat#171011; RRID:AB_2891275	1 mg/mL	1:200
Mouse monoclonal anti-βII Spectrin	BD Biosciences	Cat#612562; RRID:AB_399853	250 µg/mL	1:200
Rabbit polyclonal anti-Clathrin heavy chain	Abcam	Cat#ab21679; RRID:AB_2083165	0.8 - 1 mg/mL	1:275
sdAB anti-Synaptotagmin1	Nanotag	Cat#N2302	10 µM	1:200
sdAB anti-PSD95	Nanotag	Cat#N3705	3.5 µM	1:150
Rabbit polyclonal anti-Golga5	Sigma	Cat#HPA000892-100UL	0.1 mg/mL	1:200
Lifeact[1]	MPI Biochemistry Core Facility	-	10 µM	1:20000

Supplementary Table 7 | Experimental parameters for 13-plex neuronal atlas

Target No.	Target name	Species	1^{ry} AB/NB dilution	2^{ry} NB dilution	Imager	Imager Conc. (pM)	Incubation
1	Golga5	Rabbit	1:200	1:200	R4	150	1^{ry} + 2^{ry} o.N.
2	βII Spectrin	Mouse	1:200	1:200	L1	50	1^{ry} + 2^{ry} o.N.
3	Gephyrin	Mouse	1:200	1:200	L3	400	Pre-incubation
4	PSD95	direct	1:150	-	R2	150	Pre-incubation
5	VGAT	Rabbit	1:250	1:150	R5	150	Pre-incubation
6	TOM20	Rabbit	1:200	1:200	R6	150	Pre-incubation
7	NeurofilL	Mouse	1:200	1:200	R1	50	Pre-incubation
8	VGlut1	Rabbit	1:200	1:200	L2	50	Pre-incubation
9	Synaptotagmin1	direct	1:200	-	R3	50	Pre-incubation
10	Pmp70	Rabbit	1:200	1:200	L4	100	Pre-incubation
11	Clathrin HC	Rabbit	1:275	1:200	L5	150	Pre-incubation
12	Bassoon	Mouse	1:200	1:200	L6	150	Pre-incubation
13	Actin	-	-	-	Lifeact	500	-

Supplementary Table 8 | Localization precision for 13-plex neuronal atlas

Protein Target	Loc precision (nm) Pos0	Loc precision (nm) Pos1	Loc precision (nm) Pos2	Loc precision (nm) Pos3
Golga5	4.78	4.94	4.8	5.17
βII Spectrin	5.3	5.93	5.61	5.43
Gephyrin	6.26	6.61	6.4	6.28
PSD95	4.37	5.22	5.18	5.11
VGAT	4.7	5.27	4.72	5.03
Tom20	4.16	4.48	4.17	4.44
Neurofilament L	3.87	4.48	3.99	4.52
VGlut1	4.16	4.61	4.76	4.42
Synaptotagmin1	4.19	4.69	4.79	4.57
Pmp70	4.58	4.43	4.84	4.68
Clathrin Heavy Chain	5.19	5.66	5.81	5.56
Bassoon	4.51	5.17	4.95	4.85
LifeAct	5.56	6.3	6.84	5.95

Supplementary Table 9 | Comparison of current multiplexing approaches

Multiplexing approach	Highest reported k_{on}	Use of secondary adapters	Maximal number of targets achieved	Example imaging time for single target (comparable resolution and sampling)	Additional time for barcoding	Theoretical time for 13-plex neuronal atlas
Combined left and right-handed speed-optimized Exchange-PAINT	$81.2 * 10^6 (Ms)^{-1}$	no	13	~ 10 min	-	~ 10 h
SUM-PAINT Unterauer et al. (2024)[2]	$62.1 * 10^6 (Ms)^{-1}$	yes	30 (theoretically unlimited)	~ 10 min	30 min per 6 targets	~ 10:30 h
FLASH-PAINT Schueder et al. (2024)[3]	$60.3 * 10^6 (Ms)^{-1}$	yes	13 (theoretically unlimited)	~ 10 min	-	~ 10 h
Speed-optimized Exchange-PAINT Strauss et al. (2020)[4]	$77 * 10^6 (Ms)^{-1}$	no	6	~ 10 min	-	-
Exchange-PAINT Jungmann et al. (2014)[5]	$1.25 *10^6 (Ms)^{-1}$	no	52 (theoretically unlimited)	~ 650 min	-	650 h

Supplementary Table 10 | Imaging parameters

Dataset	Parameters	Buffer	Power (561 nm)
**Figure 1b**	75 ms, 15k Frames, 100 pM for R1, R3 (long), R4, R6, L1, L2 – 150 pM for R5 – 200 pM for R2, L4, L6 – 500 pM for L3 and L5	C+	50 mW – 250 W/cm²
**Figure 1c**	100 ms, 60k Frames for Tom20 and Nup96-GFP -120k Frames for α-Tubulin, 50 pM L4 (Tom20) – 25 pM L1 (α-Tubulin) – 100 pM L2 (Nup96-GFP)	C+	18 mW – 90 W/cm²
**Figure 1d & Supplementary Figure 2**	75 ms, 15k Frames, 100 pM for R1, R4, R6, L1, L2 – 150 pM for R5 – 200 pM for R2, L4, L6 – 400 pM for R3 (short) – 500 pM for L3 and L5	C+	50 mW – 250 W/cm²
**Figure 1e & Supplementary Figure 3**	100 ms, 40k Frames, 100 pM for R1-R6 and L1-L6	C+	20 mW – 100 W/cm²
**Figure 2 & Supplementary Figure 4-7**	75 ms, 7.5k Frames, Imager concentration according to Supplementary Table 7	C+	16 mW – 80 W/cm²
**Supplementary Figure 1**	100 ms, 100k Frames for L6 – 120k Frames for L1, 25 pM for L1 – 75 pM for L6	C+	60 mW – 300 W/cm²

References

1 Riedl, J. *et al.* Lifeact: a versatile marker to visualize F-actin. *Nat Methods* **5**, 605-607 (2008). https://doi.org/10.1038/nmeth.1220
2 Unterauer, E. M. *et al.* Spatial proteomics in neurons at single-protein resolution. *Cell* **187**, 1785-1800 e1716 (2024). https://doi.org/10.1016/j.cell.2024.02.045
3 Schueder, F. *et al.* Unraveling cellular complexity with transient adapters in highly multiplexed super-resolution imaging. *Cell* **187**, 1769-1784 e1718 (2024). https://doi.org/10.1016/j.cell.2024.02.033
4 Strauss, S. & Jungmann, R. Up to 100-fold speed-up and multiplexing in optimized DNA-PAINT. *Nat Methods* **17**, 789-791 (2020). https://doi.org/10.1038/s41592-020-0869-x
5 Jungmann, R. *et al.* Multiplexed 3D cellular super-resolution imaging with DNA-PAINT and Exchange-PAINT. *Nat Methods* **11**, 313-318 (2014). https://doi.org/10.1038/nmeth.2835
