# Supplementary material for: Left-handed DNA for efficient highly multiplexed imaging at single-protein resolution
Source: Nat Commun. 2025 Oct 2;16:8773. doi: 10.1038/s41467-025-64228-x (PMC12491495; doi:10.1038/s41467-025-64228-x)
Supplement: Supplementary file 2 — Reporting Summary [file 41467_2025_64228_MOESM2_ESM.pdf]

nature portfolio

Corresponding author(s): Luciano Masullo and Ralf Jungmann

Last updated by author(s): Sep 1, 2025

Reporting Summary

Nature Portfolio wishes to improve the reproducibility of the work that we publish. This form provides structure for consistency and transparency in reporting. For further information on Nature Portfolio policies, see our Editorial Policies and the Editorial Policy Checklist.

Statistics

For all statistical analyses, confirm that the following items are present in the figure legend, table legend, main text, or Methods section.

n/a	Confirmed	
☐	☒	The exact sample size (*n*) for each experimental group/condition, given as a discrete number and unit of measurement
☐	☒	A statement on whether measurements were taken from distinct samples or whether the same sample was measured repeatedly
☒	☐	The statistical test(s) used AND whether they are one- or two-sided<br>*Only common tests should be described solely by name; describe more complex techniques in the Methods section.*
☒	☐	A description of all covariates tested
☒	☐	A description of any assumptions or corrections, such as tests of normality and adjustment for multiple comparisons
☐	☒	A full description of the statistical parameters including central tendency (e.g. means) or other basic estimates (e.g. regression coefficient) AND variation (e.g. standard deviation) or associated estimates of uncertainty (e.g. confidence intervals)
☒	☐	For null hypothesis testing, the test statistic (e.g. F, t, r) with confidence intervals, effect sizes, degrees of freedom and P value noted<br>*Give P values as exact values whenever suitable.*
☒	☐	For Bayesian analysis, information on the choice of priors and Markov chain Monte Carlo settings
☒	☐	For hierarchical and complex designs, identification of the appropriate level for tests and full reporting of outcomes
☒	☐	Estimates of effect sizes (e.g. Cohen's *d*, Pearson's *r*), indicating how they were calculated

Our web collection on statistics for biologists contains articles on many of the points above.

Software and code

Policy information about availability of computer code

Data collection	Raw microscopy data was acquired using µManager (Version 4.0) (Edelstein, A., Amodaj, N., Hoover, K., Vale, R. $ Stuurman, N. Curr. Protoc. Mol. Biol. 14.20 (2010))
Data analysis	The custom software analysis package "Picasso" was used to process raw super-resolution data. "Picasso" is open source and is described in detail in Nature Protocols volume 12, pages 1198-1228 (2017) doi:10.1038/nprot.2017.024<br>The python packages seaborn, pandas, matplotlib and scipy were used to for data fitting and plotting.

For manuscripts utilizing custom algorithms or software that are central to the research but not yet described in published literature, software must be made available to editors and reviewers. We strongly encourage code deposition in a community repository (e.g. GitHub). See the Nature Portfolio guidelines for submitting code & software for further information.

Data

Policy information about availability of data

All manuscripts must include a data availability statement. This statement should provide the following information, where applicable:

- Accession codes, unique identifiers, or web links for publicly available datasets
- A description of any restrictions on data availability
- For clinical datasets or third party data, please ensure that the statement adheres to our policy

All raw data are available upon request from the authors.

Research involving human participants, their data, or biological material

Policy information about studies with human participants or human data. See also policy information about sex, gender (identity/presentation), and sexual orientation and race, ethnicity and racism.

Reporting on sex and gender	N/A
Reporting on race, ethnicity, or other socially relevant groupings	N/A
Population characteristics	N/A
Recruitment	N/A
Ethics oversight	N/A

Note that full information on the approval of the study protocol must also be provided in the manuscript.

Field-specific reporting

Please select the one below that is the best fit for your research. If you are not sure, read the appropriate sections before making your selection.

- [x] Life sciences
- [ ] Behavioural & social sciences
- [ ] Ecological, evolutionary & environmental sciences

For a reference copy of the document with all sections, see nature.com/documents/nr-reporting-summary-flat.pdf

Life sciences study design

All studies must disclose on these points even when the disclosure is negative.

Sample size	The sample size for quantitative DNA origami and nuclear pore measurements was defined by the density of the respective structures on the coverslip and the field of view chosen for acquisition.
Data exclusions	No data was excluded
Replication	All replications were successful
Randomization	Randomization was not necessary since no grouping of experiments or samples was performed
Blinding	Blinding is not applicable since no grouping was performed

Reporting for specific materials, systems and methods

We require information from authors about some types of materials, experimental systems and methods used in many studies. Here, indicate whether each material, system or method listed is relevant to your study. If you are not sure if a list item applies to your research, read the appropriate section before selecting a response.

Materials & experimental systems

n/a	Involved in the study	
[ ]	[x]	Antibodies
[ ]	[x]	Eukaryotic cell lines
[x]	[ ]	Palaeontology and archaeology
[ ]	[x]	Animals and other organisms
[x]	[ ]	Clinical data
[x]	[ ]	Dual use research of concern
[x]	[ ]	Plants

Methods

n/a	Involved in the study	
[x]	[ ]	ChIP-seq
[x]	[ ]	Flow cytometry
[x]	[ ]	MRI-based neuroimaging

Antibodies

Antibodies used	GFP Nanotag Cat# N0301 5 µM 1:200<br>Rabbit IgG Nanotag Cat# N2405 5 µM -<br>Mouse kappa light chain Nanotag Cat# N1205 5 µM -<br>Mouse monoclonal anti- α-Tubulin Sigma-Aldrich Cat# T6199;<br>RRID:AB_477583 1.0 -1.2 mg/mL 1:200<br>Mouse monoclonal anti-Bassoon Enzo Life Sciences Cat# ADI-VAM-PS003-F;

	RRID:AB_1118105 1 mg/mL 1:200<br>Mouse monoclonal anti-Gephyrin Synaptic Systems Cat#147 011;<br>RRID:AB_887717 1 mg/mL 1:200<br>Rabbit polyclonal anti-VGAT invitrogen Cat#PA5-27569;<br>RRID:AB_2545045 1.46 mg/mL 1:250<br>Rabbit monoclonal anti-Tom20 Abcam Cat#ab186735;<br>RRID:AB_2889972 0.94 mg/mL 1:200<br>Rabbit polyclonal anti-VGlut1 Synaptic Systems Cat#135303;<br>RRID:AB_887875 1 mg/mL 1:200<br>Rabbit polyclonal anti-PMP70 Abcam Cat#ab85550;<br>RRID:AB_10672335 0.8 - 1 mg/mL 1:200<br>Mouse monoclonal anti-Neurofilament L Synaptic Systems Cat#171011;<br>RRID:AB_2891275 1 mg/mL 1:200<br>Mouse monoclonal anti-βII Spectrin BD Biosciences Cat#612562;<br>RRID:AB_399853 250 µg/mL 1:200<br>Rabbit polyclonal anti-Clathrin heavy chain Abcam Cat#ab21679;<br>RRID:A_2083165 0.8 - 1 mg/mL 1:275<br>sdAB anti-Synaptotagmin1 Nanotag Cat#N2302 10 µM 1:200<br>sdAB anti-PSD95 Nanotag Cat#N3705 3.5 µM 1:150<br>Rabbit polyclonal anti-Golga5 Sigma Cat#HPA000892-100UL 0.1 mg/mL 1:200
Validation	All antibodies were validated for IF and respective species reactivity by the manufacturer according to their websites.

Eukaryotic cell lines

Policy information about cell lines and Sex and Gender in Research

Cell line source(s)	U2OS-Nup96-mEGFP cells were obtained from the Ellenberg and Ries lab (Reference: doi.org/10.1038/s41592-019-0674-9.
Authentication	The cell lines were not authenticated
Mycoplasma contamination	All cell lines have been tested negative for mycoplasma contamination
Commonly misidentified lines<br>(See ICLAC register)	No commonly misidentified cell lines were used

Animals and other research organisms

Policy information about studies involving animals; ARRIVE guidelines recommended for reporting animal research, and Sex and Gender in Research

Laboratory animals	Wild-type Wistar rat pregnant mothers or pups (Rattus norvegicus) and adult mice (Mus musculus) were obtained from the University Medical Center Göttingen
Wild animals	N/A
Reporting on sex	Animals of both sexes were used in this study, as cultures were prepared from male and female rat embryos in a 1:1 ratio, and mouse brain slices were obtained from both male and female mice.
Field-collected samples	Animals were not used in other studies, maintained on a standard chow diet, kept at room temperature under a constant light cycle, and screened regularly for common rodent viruses and pathogens. Embryos were obtained from pregnant female rats not used in other studies
Ethics oversight	Animals were handled according to the specifications of the University of Göttingen and of the local authority, the State of Lower Saxony (Landesamt für Verbraucherschutz, LAVES, Braunschweig, Germany). Animal experiments were approved by the local authority, the Lower Saxony State Office for Consumer Protection and Food Safety (Niedersächsisches Landesamt für Verbraucherschutz und Lebensmittelsicherheit).

Note that full information on the approval of the study protocol must also be provided in the manuscript.

Plants

Seed stocks	N/A
Novel plant genotypes	N/A
Authentication	N/A
